# Incidence and survival of transthyretin amyloid cardiomyopathy from a French nationwide study of in- and out-patient databases

**DOI:** 10.1186/s13023-023-02933-w

**Published:** 2023-11-06

**Authors:** Thibaud Damy, Guillaume Bourel, Michel Slama, Vincent Algalarrondo, Olivier Lairez, Pauline Fournier, Jérôme Costa, Françoise Pelcot, Agnès Farrugia, Isabelle Durand Zaleski, Hervé Lilliu, Caroline Rault, Mathilde Bartoli, Stéphane Fievez, Anna Granghaud, Jeremie Rudant, Agathe Coste, Charlotte Noirot Cosson, Pierre-Alexandre Squara, Marion Narbeburu, Bertrand De Neuville, Philippe Charron

**Affiliations:** 1 Referral Center for Cardiac Amyloidosis, Mondor Amyloidosis Network, GRC Amyloid Research Institute and Cardiology Department, APHP Henri Mondor Hospital, Créteil, France; 2 NSERM Unit U955, Team 8, Paris-Est Creteil University, Créteil, France; 3 Accenture, Paris, France; 4 Competence Center for Cardiac Amyloidosis, APHP Bichat Hospital, Cardiology Department, CRMR NNERF, Paris, France; 5 Department of Cardiology, Rangueil University Hospital, Toulouse, France; 6 Department of Cardiology, Reims University Hospital, Reims, France; 7 Association Française Contre L’Amylose, Marseille, France; 8 Paris University, CRESS, INSERM, INRA, AP-HP, Public Health Henri Mondor Hospital & URCEco, Hotel Dieu Hospital, 75004 Paris, France; 9 Inbeeo, London, England; 10 DataGnosis, Rennes, France; 11 Pfizer, Paris, France; 12 APHP, Department of Genetics & Department of Cardiology, Referral Center for Hereditary or Rare Cardiac Diseases, Pitié-Salpêtrière Hospital, Paris, France; 13 Sorbonne Université, INSERM, UMR_S 1166 and ICAN Institute for Cardiometabolism and Nutrition, Paris, France

**Keywords:** Amyloidosis, Heart failure, Transthyretin, Incidence, Survival, Real world data

## Abstract

**Background:**

Precise data about ATTR-CM incidence rates at national level are scarce. Consequently, this study aimed to estimate the annual incidence and survival of transthyretin amyloid cardiomyopathy (ATTR-CM) in France between 2011 and 2019 using real world data. We used the French nationwide exhaustive data (*SNDS* database) gathering in- and out-patient claims. As there is no specific ICD-10 marker code for ATTR-CM, diagnosis required both amyloidosis (identified by E85. ICD-10 code or a tafamidis meglumine delivery) and a cardiovascular condition (identified by ICD-10 or medical procedure codes related to either heart failure, arrhythmias, conduction disorders or cardiomyopathies), not necessarily reported at the same visit. Patients with probable AL-form of amyloidosis or probable AA-form of amyloidosis were excluded.

**Results:**

Between 2011 and 2019, 8,950 patients with incident ATTR-CM were identified. Incidence rates increased from 0.6 / 100,000 person-years in 2011 to 3.6 / 100,000 person-years in 2019 (*p* < 0.001), reaching 2377 new cases in 2019. Sex ratios (M/F) increased from 1.52 in 2011 to 2.23 in 2019. In 2019, median age at diagnosis was 84.0 years (85.5 for women and 83.5 for men). Median survival after diagnosis was 41.9 months (95% CI [39.6, 44.1]).

**Conclusions:**

This is the first estimate of nationwide ATTR-CM incidence in France using comprehensive real-world databases. We observed an increased incidence over the study period, consistent with an improvement in ATTR-CM diagnosis in recent years.

## Background

Amyloidosis is a group of rare conditions in which amyloid fibrils accumulate in various organ tissues, leading to failure of the affected organ. There are several histological forms of amyloidosis: transthyretin (TTR) amyloidosis (ATTR) and amyloid light-chain (AL) amyloidosis are the most frequent [1]. TTR amyloidosis phenotype can be predominantly cardiac, neuropathic, or mixed. When affecting the heart, amyloid TTR protein fibrils can lead to transthyretin amyloid cardiomyopathy (ATTR-CM). The resulting restrictive cardiomyopathy can cause congestive heart failure leading to death.

ATTR-CM is either hereditary (ATTRv-CM) or wild type (ATTRwt-CM, formerly called senile ATTR-CM) [2, 3].

ATTRwt-CM is an underdiagnosed disease [4] that accounts for approximately 13% [5] of heart failure (HF) with preserved ejection fraction cases. Therefore, targeting ATTR-CM screening in the presence of HF symptoms based on risk factors for the wild type form (male, older than 60) [6–9] or associated clinical conditions (including carpal tunnel syndrome, valvulopathies, [10], conduction disorders, arrhythmias as atrial fibrillation, polyneuropathy [11], among others) may identify the majority of ATTR-CM in western countries. Hereditary forms may start before the age of 60.

Comorbidity and mortality associated with ATTR-CM represent a high health burden for patients. Health-related quality of life is severely affected and declines as the disease progresses [12–14].

Studies show median survival varying from 26 to 70 months after diagnosis [15–17].

In recent years, ATTR has received increased attention from the medical community due to an improved knowledge of the disease and the availability of tafamidis meglumine 20 mg to treat the neurologic form of the disease. More recently, diagnosis has improved significantly, especially with the use of bone scintigraphy, which can diagnose ATTR-CM without the need for a heart biopsy [18]. Since November 2018, the availability of tafamidis meglumine 20 mg for ATTR-CM patients through early access programs offers a therapeutic option for these patients [19] who previously had no disease-specific medicinal treatments available.

Precise data about ATTR-CM incidence rates at national level are scarce. Consequently, this study aimed to estimate the French incidence of ATTR-CM, to describe the variation in these trends by age and gender and to estimate survival of ATTR-CM patients.

## Methods

### Data source

Healthcare insurance is compulsory in France, providing single-payer national universal coverage for the population (over 67 million people). Its information system relies on several databases which collect exhaustively demographics, city of residence, healthcare professional (HCP) visits, drugs, medical devices, lab test and imagery prescriptions (not the results), hospital admissions and home hospitalizations, including cause of hospitalization (ICD-10, International Classification of Diseases, release 10), medical acts, and resulting diagnosis-related groups, and long-term conditions associated with full coverage of expenditures (ALD: *Affections de Longue Durée*). Database are consolidated at the individual level resulting in the SNDS (*Système National des Données de Santé*). The SNDS has been recognized as a powerful source of information for pharmacoepidemiology studies [20].

Medical codes used were ICD-10 for diagnosis and ALD, ATC (Anatomical Therapeutic Chemical), UCD (*Unité Commune de Dispensation*: common dispensing unit), and CIP (*Code d’Identification de Présentation*: medication package classification), the last two being French specific codes for drugs, and CCAM (*Classification Commune des Actes Médicaux*) for HCP prescriptions / medical acts.

## Experimental design

**Fig. 1:**
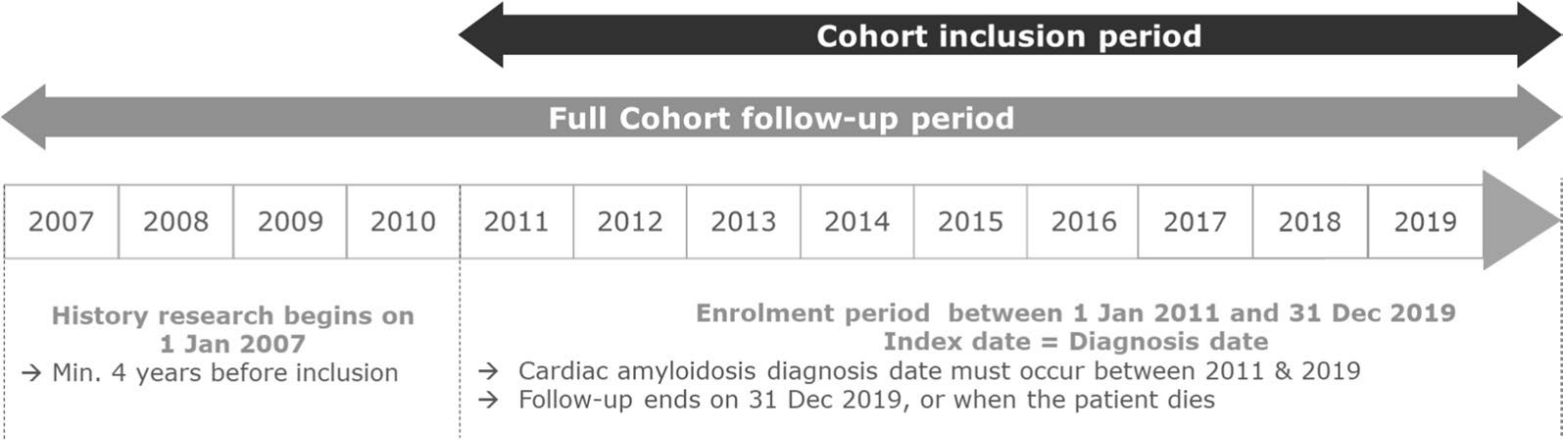
Study design

We designed a retrospective longitudinal nationally exhaustive analysis of SNDS claims data. Using this database, ATTR-CM patients were identified from January 1, 2011 to December 31, 2019 (last available year at the time of data analysis). End of follow-up was either December 31, 2019 or death. The years 2007–2010 were used to document pre-diagnosis medical anamnesis (including the first code of either cardiac condition or amyloidosis) and to exclude prevalent cases (those with both cardiac conditions and amyloidosis before 2011). Pre-2007 data were not used because of known lower quality. Figure 1 describes the study design.

## Variable definition

As there is no specific ICD-10 code for ATTR-CM used in the SNDS, ATTR-CM diagnosis required both an amyloidosis (identified by ICD-10 E85 or a tafamidis meglumine delivery) and a related cardiovascular condition (identified by ICD-10 or medical procedure codes related to heart failure, arrhythmia, conduction disorder or cardiomyopathy) (list of codes available on request from the corresponding author / provided as a supplementary table, as chosen by the editor). As both conditions are chronic, they did not need to be identified during the same medical event, and no upper limit delays were fixed between them. Index diagnosis date referred to the last of the first pair (among amyloidosis and cardiovascular conditions) of selected reported conditions or to the first delivery of tafamidis meglumine prescribed by a cardiologist. Hospital admission, entry date into the ALD regime or delivery date of tafamidis meglumine was considered. Date of death was the one reported in the SNDS database.

## Data extraction of amyloidosis patients from the SNDS

Comprehensive data on all patients with at least one ICD-10 diagnosis code for amyloidosis (i.e., E85) during an hospital stay or an ALD status reported between January 1, 2007 and December 31, 2019 or at least one delivery of tafamidis meglumine between January 1, 2011 (tafamidis meglumine started to be dispensed in an early access program in 2011) and December 31, 2019 were extracted by the national health Insurance (CNAM) who holds the full database. Data extraction encompassed claims from January 1, 2007 to December 31, 2019.

## Patient selection for ATTR

**Fig. 2:**
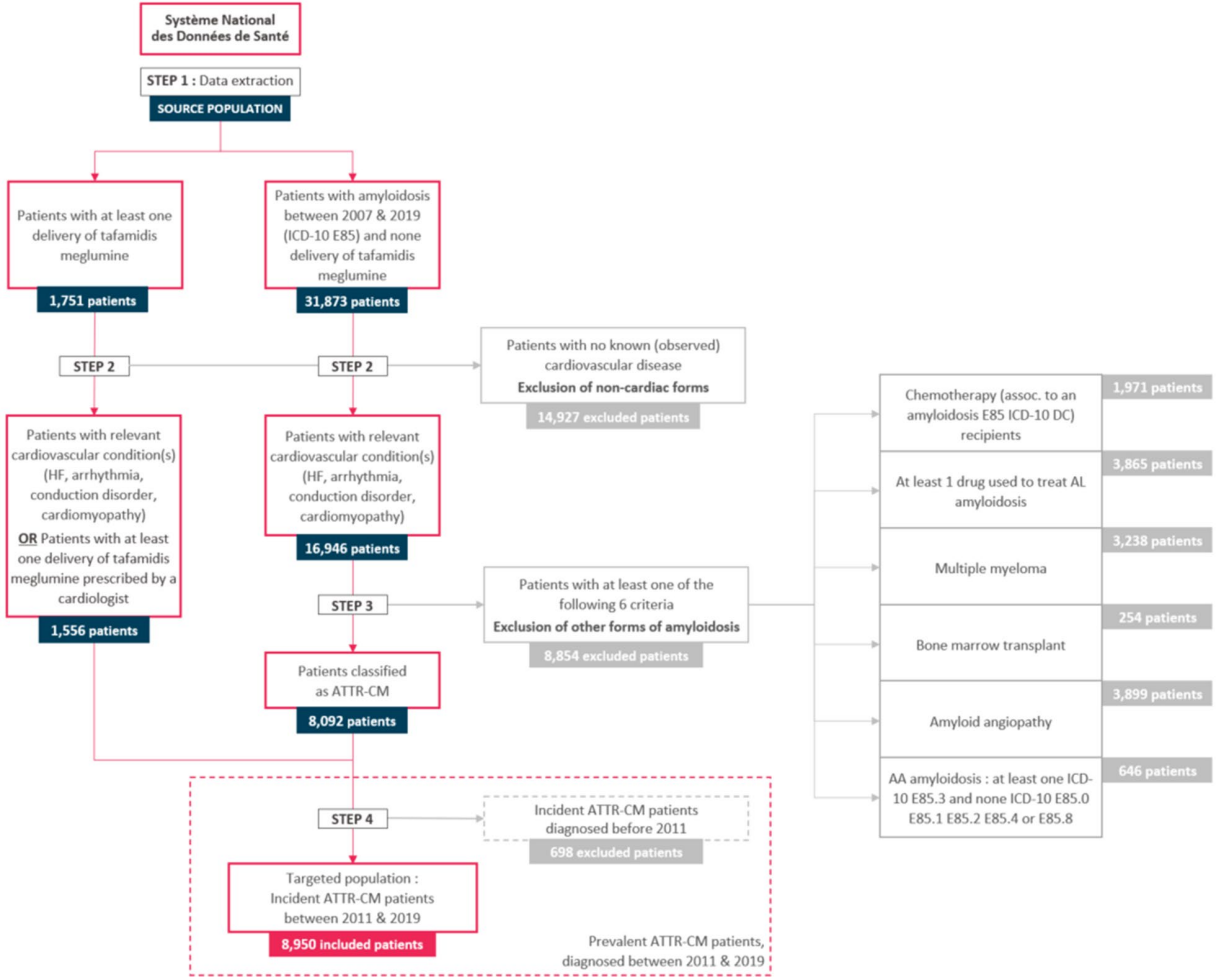
Population flowchart HF: Heart Failure. ICD-10: International Classification of Disease, release 10. ATTR-CM: Transthyretin amyloid cardiomyopathy. AL: Amyloid light-chain

An algorithm was developed to identify ATTR-CM patients, as described in Fig. 2, whatever their age and sex and wild-type or hereditary form. It was run on the extracted dataset, to select patients with ATTR-CM from the initial pool of amyloidosis patients.

As described below, ATTR-CM patients were selected through two approaches: on one side, patients who received tafamidis meglumine delivery; on the other side, patients without tafamidis meglumine, selected through clinical conditions.

For all, cardiac conditions were identified by ICD-10 or CCAM codes during at least one hospital stay or an ALD status related either to heart failure, arrhythmias, conduction disorders or cardiomyopathies.

On one side, as the consumption of tafamidis meglumine is associated with transthyretin amyloidosis condition, we identified all patients with at least one tafamidis meglumine delivery and at least one of the selected cardiac conditions (heart failure, arrhythmias, conduction disorders, cardiomyopathies) or at least one tafamidis meglumine delivery prescribed by a cardiologist as ATTR-CM patients.

On the other side, for all patients with an E85 ICD-10 diagnosis code and no tafamidis meglumine delivery, we applied selection and exclusion criteria to identify ATTR-CM patients. Patients had to have at least one of the selected cardiac conditions (heart failure, arrhythmias, conduction disorders, cardiomyopathies).

Amyloid light-chain (AL) amyloidosis patients were excluded based on the delivery of any of the following medications (identified by their CIP and UCD codes): bendamustine, daratumumab, lenalidomide, thalidomide, bortezomib, carfilzomib, and pomalidomide, used against AL amyloidosis form with plasma cell proliferation, and venetoclax, ibrutinib, rituximab and obinutuzumab, used against AL amyloidosis form with lymphocyte proliferation. Cyclophosphamide and melphalan were not used to exclude AL amyloidosis patients as these drugs, only dispensed in hospital and being inexpensive, are not tracked while dispensed during hospital stays.

Patients with AA amyloidosis, i.e., with at least one E85.3 ICD-10 (secondary systemic amyloidosis) and with no E85.0 (non-neuropathic heredofamilial amyloidosis), E85.1 (neuropathic heredofamilial amyloidosis), E85.2 (heredofamilial amyloidosis, unspecified), E85.4 (organ-limited amyloidosis) or E85.8 (other amyloidosis), were excluded.

Patients who received chemotherapy for amyloidosis, i.e., Z51.1 (chemotherapy session for neoplasm) or Z51.2 (other chemotherapy) associated with an E85 ICD-10 diagnosis code during the same chemotherapy session, were excluded.

Patients with multiple myeloma (C90.0 ICD-10), bone marrow transplant (Z94.80 ICD-10), or a history of amyloid angiopathy (I68.0 ICD-10), generally associated with Alzheimer’s disease, were also excluded.

Individuals diagnosed between 2007 and 2010 were excluded to select only incident cases.

## Statistical analysis

This was an analysis based on a national exhaustive database and therefore no inferential statistics were produced. Qualitative variables were described by the documented frequencies and percentages of each modality. Quantitative variables were described by the documented frequency, mean, median, quartiles, and extreme values.

Annual incidence rate was reported as the annual number of newly diagnosed cases, over the year-matched number of French citizens alive in the same year, based on INSEE (French National Institute of Statistics and Economic Studies) data [21].

Since ATTR-CM was a rare disease, a proxy method [22] was used:

$$\mathrm{Person}-\mathrm{years}=\frac{{\mathrm{N}}_{\mathrm{i}}+{\mathrm{N}}_{\mathrm{i}+1}}{2}{\mathrm{x }\Delta }_{\mathrm{t}}$$; Where:

N_i_ = Size of the French population for year i.

N_(i+1)_ = Size of the French population for year i + 1.

Δt = Time between year i and i + 1 expressed in years.

Cochran–Armitage trend test was used to assess evolution of annual incidence over time. P-values less than 0.05 were considered statistically significant.

Time from diagnosis to death was described using Kaplan–Meier’s method, death being the event and December 31, 2019 the censoring date. Estimates were median, mean, interquartile range, minimum, and maximum survival in months, rates of survival at one and three years after the diagnosis, and gross number of deaths during the follow-up period (frequency and proportion).

Age (< 50; [50–59]; [60–69]; [70–79]; [80–89]; ≥ 90) at the index date and sex were used as subgroups of interest.

## Results

Data from 33,624 patients were extracted using the E85 amyloidosis diagnosis code or tafamidis meglumine delivery over the study period. The patient selection algorithm presented above was applied to this initial dataset to identify ATTR-CM patients.

Among the 1,751 patients treated by tafamidis meglumine, 1,556 (88.9%) had at least one ICD-10 diagnosis code associated with cardiac disease or a delivery of tafamidis meglumine prescribed by a cardiologist. These patients were identified as ATTR-CM patients.

Among the 31,873 patients with no tafamidis meglumine delivery and at least one ICD-10 E85, 16,946 (53.2%) had at least one ICD-10 diagnosis or procedural code associated with a cardiac disease. 8,854 were excluded for the following reasons (exclusion reasons are not mutually exclusive): drug delivery to treat AL amyloidosis (3865, 43.7%), chemotherapy related to amyloidosis (1971, 22.3%), multiple myeloma (3238, 36.6%), bone marrow transplantation (254, 2.9%), cerebral amyloid angiopathy (3899, 44.0%), and AA amyloidosis (646, 7.3%). The remaining 8092 patients were classified as ATTR-CM.

Finally, 8950 ATTR-CM patients were identified and analysed between 2011 and 2019.

Among these 8,950 ATTR-CM patients, 97% were identified with at least one ICD-10 E85 in their medical history: 91% with a related hospital stay and no related ALD status, 5% with both related hospital stay and related ALD status and 1% with only a related ALD status and no related hospital stay. Hence, the identification of amyloidosis condition through E85 ICD-10 code is widely due to hospital stays, and very few patients are identified through ALD status.

**Fig. 3:**
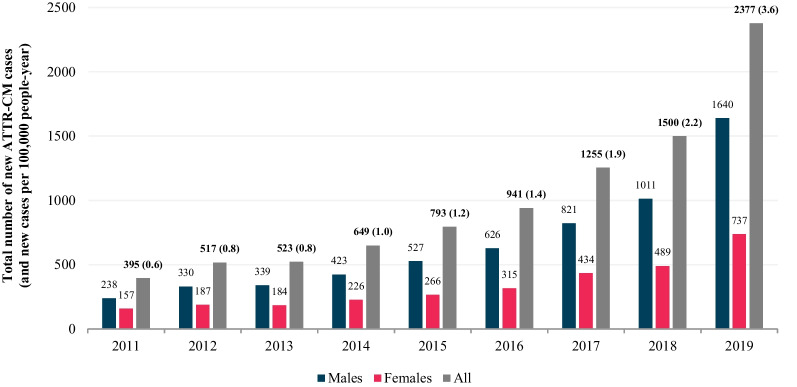
ATTR-CM incidence overall and by gender between 2011 and 2019

Figure 3 describes the ATTR-CM incidence rates from 2011 to 2019 overall and by gender.

The incidence rate multiplied over five times, from 0.6 / 100,000 person-years in 2011 to 3.6 / 100,000 person-years in 2019 (*p* < 0.001), reaching 2,377 new cases in 2019.

Moreover, sex ratios (M/F) increased from 1.52 in 2011 to 2.23 in 2019.

**Fig. 4:**
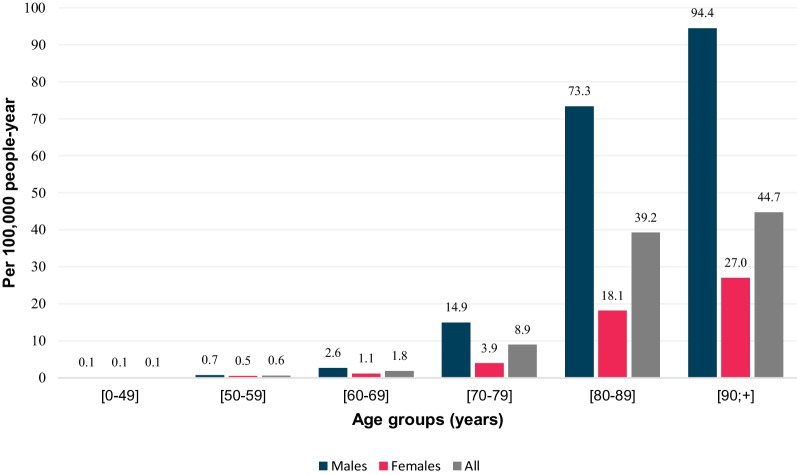
Incidence rate in 2019 by age group and gender

Figure 4 illustrates the incidence rate by age and gender, according to diagnosis in 2019.

81.7% of the selected patients were older than 70 years of age. Sex ratio was 1.99 males: 1 female for the complete cohort and 2.23 males: 1 female in 2019.

Table 1 reports on age distribution and sex ratio at diagnosis of the complete cohort and 2019 incident cases.

**Table 1:** Mean age at diagnosis, by gender and age distribution for the complete cohort and2019 incident cases

Age at diagnosis	Complete cohort			2019 incident cases		
	All	Males	Females	All	Males	Females
Mean (SD)	78.7 (12.6)	78.6 (11.8)	78.9 (14.0)	81.9 (10.3)	81.6 (9.3)	82.4 (12.4)
Median (Q1, Q3, IQR)	82.0 (73.5, 87.0, 13.5)	81.5 (73.5, 86.5, 13.0)	83.0 (73.5, 88.0, 14.5)	84.0 (78.0, 88.0, 10.0)	83.5 (77.5, 87.5, 10.0)	85.5 (79.0, 89.5, 10.5)
< 50. n (%)	330 (3.7%)	176 (3.0%)	154 (5.1%)	35 (1.5%)	18 (1.1%)	17 (2.3%)
[50–59] n (%)	398 (4.4%)	254 (4.3%)	144 (4.8%)	51 (2.1%)	30 (1.8%)	21 (2.8%)
[60–69] n (%)	910 (10.2%)	609 (10.2%)	301 (10.1%)	147 (6.2%)	100 (6.1%)	47 (6.4%)
[70–79] n (%)	2,071 (23.1%)	1,530 (25.7%)	541 (18.1%)	486 (20.4%)	370 (22.6%)	116 (15.7%)
[80–89] n (%)	4,084 (45.6%)	2,760 (46.3%)	1,324 (44.2%)	1,265 (53.2%)	904 (55.1%)	361 (49.0%)
≥ 90. n (%)	1,157 (12.9%)	626 (10.5%)	531 (17.7%)	393 (16.5%)	218 (13.3%)	175 (23.7%)
n (%)	8,950	5,955 (66.5%)	2,995 (33.5%)	2,377	1,640 (69.0%)	737 (31.0%)

SD: standard deviation. IQR: interquartile range

For the complete cohort, median age at diagnosis was 82.0 years, 83.0 for women and 81.5 for men. In 2019, median age at diagnosis was 84.0 years, 85.5 for women and 83.5 for men. Of note, women were diagnosed later (23.7% were older than 90 years) than men (13.3%), as reported in Fig. 4.

**Fig. 5:**
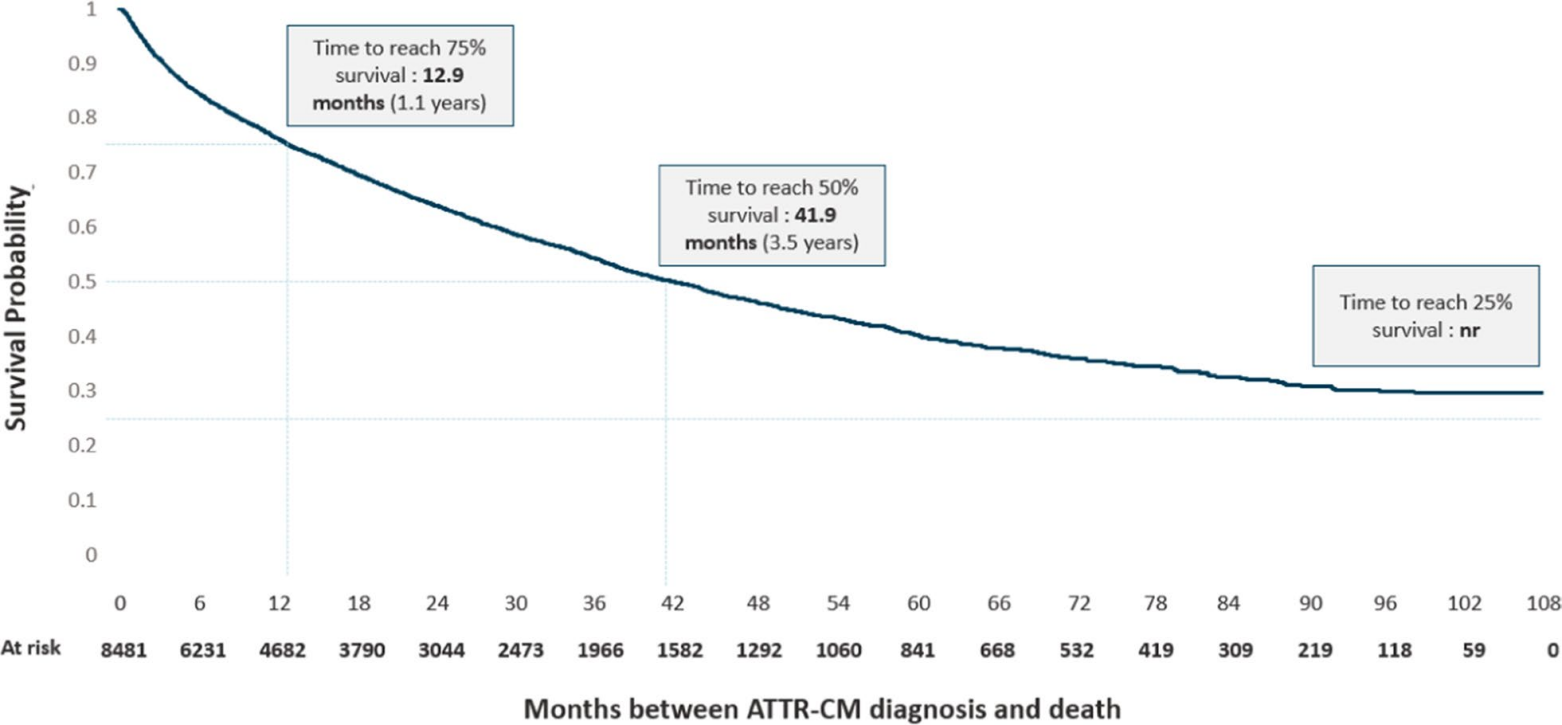
Survival curve after transthyretin amyloid cardiomyopathy diagnosis Survival analysis was performed on 8481 patients, excluding 469 patients who died during the hospital stay of their ATTR-CM diagnosis

Figure 5 describes mortality after ATTR-CM diagnosis, for all patients excluding 469 patients who died during the hospital stay of their ATTR-CM diagnosis.

Median survival was 41.9 months (approx. 3 years and 6 months) overall (95% CI [39.6, 44.1]). Survival probability was 0.76 a year after diagnosis, 0.64 two years after, 0.59 three years after, 0.54 four years after, and 0.46 after five years

## Discussion

This is the first estimate of nationwide ATTR-CM incidence rates in France. To our knowledge, only a few previous studies have estimated ATTR-CM prevalence or incidence [23–25]. Two were conducted in Japan and benefitted either from clinical data [27] or from ATTR-CM specific codes [26]. On the contrary, Lauppe et al. [25] also had to develop an algorithm combining cardiac conditions and amyloidosis diagnosis codes. They used a more restrictive approach than us to select cardiac conditions. They selected only heart failure and cardiomyopathies and not arrhythmias and conduction disorders and considered only cardiac conditions that occurred 2 years before or after amyloidosis.

Interestingly, our estimated incidence rate was multiplied over five times between the years 2011 and 2019. This may be due to improved diagnosis led by evolving diagnosis methods and better awareness, knowledge of the disease among healthcare professionals and availability of a new treatment (tafamidis meglumine) since November 2018 thanks to the early access program. Although changes in diagnostic approach probably have influenced the diagnostic yield, as well as the perspective of new therapy for cardiac amyloidosis, we think that this do not introduce significative bias regarding the goal of our study but rather constitute an explanation for the increasing incidence we observe.

Our study outlined a male predominance with 66.5% of men. Of note, the sex ratio has been reported to be close to 1:1 in ATTRv-CM [26] while more males are affected in ATTRwt-CM [27, 28]. With ATTRwt-CM being far more frequent than ATTRv-CM, males are more prone to ATTR-CM all forms combined, as observed in this study. In addition, the imbalanced sex ratio might be partially explained by missed diagnoses of cardiovascular disease among older women [18].

Our study also confirmed the poor prognosis of ATTR-CM patients reported in previous studies, as the median survival in our analysis (42 months) was consistent with available published data. Pinney et al. [16] reported a median survival of 2.7 years from diagnosis based on biopsy-proven ATTRwt-CM patients in 2013. Lane et al. [12] reported a median survival of 57 months among ATTRwt-CM patients. Damy et al. [29], based on a French sample, reported a median survival of 26 months.

Our study presents some limitations. There are neither specific ICD-10 ATTR-CM codes nor CCAM specific medical acts for diagnosing / treating ATTR-CM in the SNDS, which would have made the search algorithm both more sensitive and more specific. We hypothesized that all patients with amyloidosis and one or more of a list of cardiac conditions were possibly suffering from ATTR-CM. We identified several reasons limiting our estimates. First, although considered broad by the medical co-authors, this list does not guarantee that ATTR-CM patients were not incorrectly excluded from the cohort. Secondly, all patients with medical care leading us to suspect AL- or AA-amyloidosis were excluded using surrogate endpoints, assuming that the coexistence of both diseases is very rare [30, 31]. Under-reported patients with ATTR-CM if coexisting with AL- or AA- amyloidosis is still possible but marginal, so we chose a robust method to avoid over diagnosis rather than under diagnosis. Thirdly, although possible, we assumed that cases of amyloidosis with incidental cardiac expression at this age, not necessarily related to cardiac amyloidosis, is very rare once AL amyloidosis and AA-amyloidosis have been excluded. Finally, as ATTR-CM patients were identified using ICD-10 codes, comorbidities, and treatment, we cannot exclude that some rare cases of amyloidosis such as Apo A2, Apo A4 or Fibrinogen were included in the analysis. Therefore, these types of amyloidosis are very rare and should not have changed the results and conclusion of this work. Moreover, no discrimination could be made between ATTRv hereditary (ATTRv-CM) or wild type (ATTRwt-CM) with the data available, but hereditary forms are probably less represented as suggested by recent French data [32]: out of the 1208 patients who had a genetic test result available at the time of initial tafamidis prescription, 995 (82.4%) were affected with the wild-type form and 213 (17.6%) with the hereditary form.

Furthermore, the identification of ATTR-CM patients in our study is closely related to the use of E85 ICD-10 diagnosis code identifying amyloidosis condition. Except patients recently treated by tafamidis meglumine, we do not have any other sign in the SNDS claims database to detect amyloidosis condition. Hence, we missed all ATTR-CM patients without E85 ICD-10 diagnosis code and our work depends on medical practices to code E85 ICD-10. Moreover, ATTR-CM is more and more managed in ambulatory care: amyloidosis condition is almost always identified during a hospital stay with an E85 ICD-10 diagnosis code. Consequently, we assume that our index diagnosis date is probably delayed for a large part of our population, reflecting the delay between the initial clinical diagnosis and the first hospital stay with an E85 ICD-10 diagnosis code detected by our algorithm. However, we think this is reasonable estimate as ATTR-CM symptomatology leads quickly to a hospitalisation once cardiac expression has begun. Hence, it is likely that the date defined in our algorithm is close to the date of the initial diagnosis.

Looking ahead, several paths may help us better capture ATTR-CM epidemiology in the foreseeable future will allow better monitoring and better care of these patients, thus meeting European health programs goals. Further data will become available with the use of new chemical entities, which will become new markers of ATTR-CM in the SNDS. Genetic markers may be more available and accessible alongside CCAM codes, once created. Cross-validation with full medical records in a subset of patients chosen at random from the SNDS would be worthwhile to identify the reasons for and number of misclassifications, if any.

## Conclusions

To conclude, this study is the first estimate of nationwide ATTR-CM incidence in France using real-life databases. The increase in incidence over the study period suggests that ATTR-CM diagnosis is improving in Europe, likely supported by the increasing level of disease awareness in the cardiology community. Our findings may help to refine and improve public health strategies about ATTR-CM.

## Data Availability

The data that support the findings of this study are restricted by the French legislation. It prohibits the authors from making the minimal data set publicly available.
